# Cross-Campus Collaboration

**DOI:** 10.1093/geroni/igaa057.1750

**Published:** 2020-12-16

**Authors:** Claudia Oakes

**Affiliations:** University of Hartford, West Hartford, Connecticut, United States

## Abstract

This presentation will describe collaborative efforts on the campus of a mid-sized, private university to carry out activities consistent with the Age-Friendly University philosophy. In one program, staff from Career Services and a faculty member from the Department of Health Science coordinated with the President’s College (a continuing education program for adult learners), the Emeriti Association (a group of retired faculty members), and alumni to offer mock interviews for students preparing for graduate school. In another program, steps were taken to coordinate with the office of Diversity, Equity and Inclusion to address Ageism in the Workplace. The presentation will conclude with advice for identifying allies across campus and fostering support for the AFU principles.

